# Aberrant Regulation of mRNA m^6^A Modification in Cancer Development

**DOI:** 10.3390/ijms19092515

**Published:** 2018-08-25

**Authors:** Junyun Luo, Hui Liu, Siyu Luan, Chongsheng He, Zhaoyong Li

**Affiliations:** State Key Laboratory of Chemo/Biosensing and Chemometrics, College of Biology, Hunan University, Changsha 410082, China; junyluo@126.com (J.L.); liuhui4615@163.com (H.L.); luan9576@126.com (S.L.); chongshenghe@outlook.com (C.H.)

**Keywords:** m^6^A, m^6^A writer, m^6^A eraser, m^6^A reader, cancer development

## Abstract

*N*^6^-methyladenosine (m^6^A) is the most prevalent internal modification of eukaryotic messenger RNAs (mRNAs). The m^6^A modification in RNA can be catalyzed by methyltransferases, or removed by demethylases, which are termed m^6^A writers and erasers, respectively. Selective recognition and binding by distinct m^6^A reader proteins lead mRNA to divergent destinies. m^6^A has been reported to influence almost every stage of mRNA metabolism and to regulate multiple biological processes. Accumulating evidence strongly supports the correlation between aberrant cellular m^6^A level and cancer. We summarize here that deregulation of m^6^A modification, resulting from aberrant expression or function of m^6^A writers, erasers, readers or some other protein factors, is associated with carcinogenesis and cancer progression. Understanding the regulation and functional mechanism of mRNA m^6^A modification in cancer development may help in developing novel and efficient strategies for the diagnosis, prognosis and treatment of human cancers.

## 1. Introduction 

Analogous to DNA and histone, epigenetic modification to RNA species has been well documented for several decades [1]. More than 100 types of chemical modifications have been identified in native cellular RNAs, including messenger RNAs (mRNAs), ribosomal RNAs (rRNAs), transfer RNAs (tRNAs), small nuclear RNAs (snRNAs) and small nucleolar RNAs (snoRNAs) [2]. *N*^6^-methyladenosine (m^6^A) is the most abundant modification in eukaryotic mRNAs [3]. Although first discovered in 1974 [4], m^6^A modification has gotten more attention recently since the development of high-throughput sequencing. Benefiting from advancements in techniques that combine m^6^A-specific methylated RNA immunoprecipitation with high-throughput sequencing, it is possible to locate m^6^A in the transcriptome [5,6,7]. m^6^A sites are especially enriched near stop codons, in 3′-untranslated regions (3′ UTRs), and within long internal exons of mRNAs with a consensus sequence of RRACH (R corresponds to G or A; A = m^6^A; H corresponds to A, C, or U) [5,6]. *N*^6^-methyladenosine influences almost every stage of mRNA metabolism, including RNA folding and structure [8,9], maturation [10,11], nuclear export [12,13,14], translation [15,16], and decay [17,18,19], as well as other RNA modifications, such as adenosine-to-inosine editing [20]. As the most common internal mRNA modification found in eukaryotes, m^6^A modification is widely implicated in multiple biological processes, such as circadian rhythm [13], adipogenesis [11], spermatogenesis [12,21], embryonic stem cell self-renewal and differentiation [22,23,24,25], cortical neurogenesis [26], and so on. 

However, increasing evidence shows a correlation between aberrant cellular m^6^A level and cancer. Early in 1996, a direct relationship between increased m^6^A mRNA methyltransferase activity and cellular transformation was reported [27]. In 2012, it was observed that the m^6^A content varied significantly across several cancer cell lines, with relatively high levels in HepG2 and MCF7 cancer cell lines, but low levels in prostate cancer cell lines PC3 and PC9 [6], which indicates that cellular m^6^A levels of different cancer types are discrepant. More recently, some researchers have established an effective circulating tumor cell (CTC) capture system and developed a method by which DNA and RNA methylation (5-methyl-2′-deoxycytidine (5-mdC), 5-methylcytidine (5-mrC), and *N*^6^-methyladenosine (m^6^A)) could be detected by mass spectrometry in a single cell [28]. With this method, they discovered significant down-regulation of DNA 5-mdC and up-regulation of RNA 5-mrC and m^6^A in CTCs from the blood of lung cancer patients, which linked DNA and RNA methylation modification to the formation and development of cancer cells [28]. 

The effect of aberrant m^6^A level on cancer development has also been addressed. One recent study reported that m^6^A levels regulated the premature polyadenylation (pPA) that truncates the gene and affects carcinogenesis [29]. Tumor suppressor genes are frequently truncated by pPA in cancer, which leads their encoded products to be non-functional or dominant-negative. Some tumor suppressor genes, including MAGI3, LATS1, and BRCA1, undergo intronic pPA following large internal exons in breast cancer cells. One major cause is decreased m^6^A levels in these exons in pPA-activated breast cancer cells compared to untransformed mammary cells [29]. Multiple functions of RNA m^6^A methylation and their impact on cancer progression have been well reviewed elsewhere [30,31,32], and we focus here on how mRNA m^6^A modification is aberrantly regulated in cancers. We summarize the function and regulatory mechanism of mRNA m^6^A in carcinogenesis and cancer progression, which highlights m^6^A regulatory factors as potential targets for the diagnosis, prognosis and treatment of human cancers. 

## 2. m^6^A “Writers”, “Erasers” and “Readers”

**Figure 1:**
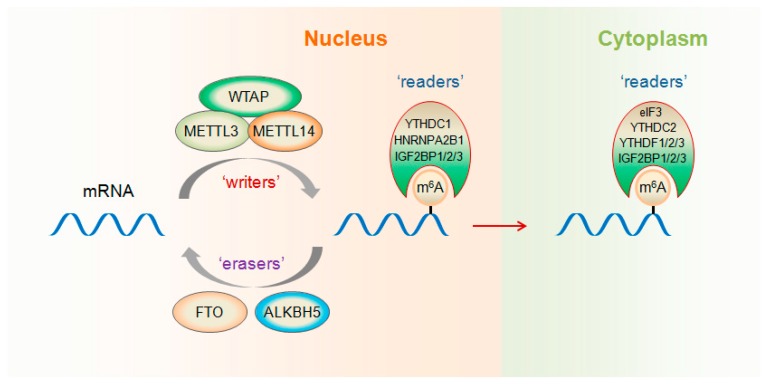
The writer, eraser, and direct reader proteins of *N*^6^-methyladenosine (m^6^A). m^6^A modification is dynamically regulated by writers (METTL3 or METTL14) and erasers (fat mass and obesity-associated (FTO) or ALKBH5), both of which are localized primarily in the nucleus. In the nucleus, m^6^A can be recognized and directly bound by m^6^A readers YTHDC1, HNRNPA2B1, andIGF2BP1/2/3. In the cytoplasm, m^6^A can be recognized and directly bound by m^6^A readers YTHDF1/2/3, YTHDC2, eIF3, and IGF2BP1/2/3. Recognition and binding of m^6^A by different readers in the nucleus or cytoplasm mediate divergent biological functions. WTAP, Wilms’ tumor 1-associating protein.

m^6^A RNA modification can be installed enzymatically by various methyltransferases, collectively termed m^6^A “writers”. The first identified human RNA m^6^A methyltransferase was METTL3. In 1997, a ~200-kDa methyltransferase complex exhibiting methyltransferase activity was isolated from HeLa cell nuclear extract, and a 70-kDa subunit containing the *S*-adenosylmethionine-binding site was identified [33]. Because of its molecular weight, this subunit was named MT-A70 (or METTL3). In 2014, METTL14 was discovered as another RNA methyltransferase, sharing 43% identity with METTL3 and forming a stable heterodimer core complex with it [34]. This METTL3–METTL14 complex functions in cellular m^6^A deposition on mammalian nuclear RNAs (Figure 1). Besides METTL3 and METTL14, Wilms’ tumor 1-associating protein (WTAP) has been identified as another component of the human m^6^A methyltransferase complex [34,35]. By interacting with METTL3 and METTL14, WTAP contributes to their localization into nuclear speckles [35]. Moreover, WTAP is necessary for the catalytic activity of m^6^A methyltransferase in vivo. Therefore, WTAP functions as a regulatory subunit in the m^6^A methyltransferase complex and promotes recruitment of the m^6^A methyltransferase complex to mRNA targets [35]. Recently, METTL16 was revealed to be another kind of human m^6^A methyltransferase that binds to pre-mRNAs and non-coding RNAs including the U6 snRNA [36]. 

Internal m^6^A modification in mammalian messenger and non-coding RNAs is dynamic and reversible. *N*^6^-methyladenosine in RNA can be removed by demethylases, which are termed m^6^A “erasers”. In 2011, fat mass and obesity-associated (FTO) protein, an obesity susceptibility factor [37], was revealed as the first RNA *N*^6^-methyladenine demethylase [38]. FTO had efficient oxidative demethylation activity to target the copious *N*^6^-methyladenosine residues in RNA in vitro and affect the amount of m^6^A in cellular RNA in vivo. Later, in 2013, ALKBH5 was discovered as another mammalian demethylase that catalyzes the removal of m^6^A modification on RNA in vitro and in vivo [12]. In addition, the demethylation activity of ALKBH5 significantly influences assembly of mRNA processing factors in nuclear speckles, mRNA export, and RNA metabolism. 

Proteins that selectively bind m^6^A can be defined as m^6^A “readers” that exert regulatory functions by selective recognition of methylated RNA. The human YTH domain family 2 (YTHDF2) specifically binds m^6^A-containing RNA as a reader protein and accelerates the decay of m^6^A-modified transcripts [17]. However, YTHDF1 selectively recognizes m^6^A-modified mRNAs and promotes ribosome occupancy of these mRNAs [15]. By interacting with initiation factors, YTHDF1 facilitates translation initiation of its mRNA targets. Although YTHDF1 and YTHDF2 share a great number of common target mRNAs, YTHDF1 binds RNA earlier during the mRNA life cycle than YTHDF2 does. Therefore, YTHDF1-mediated translation enhancement increases translation efficiency in the cytoplasm, whereas the following YTHDF2-mediated degradation controls the lifetime of target transcripts [15]. It presents a dynamic and multi-dimensional mechanism of m^6^A modification in regulating gene expression. YTHDF3, another direct reader protein of m^6^A, promotes protein synthesis through cooperation with YTHDF1 and affects methylated mRNA degradation mediated by YTHDF2 [39]. Consequently, all three YTHDF proteins may act in a synergistic manner to influence foundational biological processes related to m^6^A modification. Nevertheless, as an m^6^A reader in the nucleus, YTHDC1, has been reported to regulate mRNA splicing [10]. By recruiting pre-mRNA splicing factor SFSF3 while blocking SRSF10 mRNA binding, YTHDC1 enhances exon inclusion of targeted mRNAs. YTHDC2, the final member of the YTH protein family, has been revealed to affect the translation efficiency and abundance of its target mRNAs [21]. 

Additionally, RNA binding proteins HNRNPA2B1 and eIF3 have also been identified as direct m^6^A readers [40,41] (Figure 1). HNRNPA2B1 binds a subset of primary miRNA transcripts in the nucleus, interacts with the DGCR8 protein, a component of the pri-miRNA microprocessor complex, and facilitates primary miRNA processing [40]. The levels of m^6^A within 5′ UTRs are selectively increased by diverse cellular stresses [41]. eIF3, a component of the 43S translation preinitiation complex, directly binds to m^6^A residues within the 5′ UTRs of mRNAs and promotes cap-independent translation [41]. Recently, insulin-like growth factor 2 mRNA-binding proteins 1, 2 and 3 (IGF2BP1/2/3) have been discovered as a new family of m^6^A readers that recognize and bind m^6^A by their KH domains [42].

## 3. Function of m^6^A Writers in Cancer Development

METTL3, the first identified *S*-adenosylmethionine-binding subunit of the RNA methyltransferase complex, has been identified to play critical roles in multiple cancers [43,44,45]. METTL3 has been reported to inhibit myeloid differentiation of normal hematopoietic and leukemia cells [43]. Moreover, m^6^A modulated by METTL3 promotes leukemogenesis. METTL3 mRNA and protein expression in human acute myeloid leukemia (AML) cells are significantly higher than in healthy hematopoietic stem/progenitor cells (HSPCs) or other types of tumor cells. Furthermore, depletion of METTL3 in human myeloid leukemia cell lines facilitates differentiation and apoptosis and delays leukemia development in recipient mice in vivo. Mechanistically, METTL3 catalyzes m^6^A formation and promotes the translation of specific mRNAs critical for the regulation of proliferation, survival, and differentiation, including c-MYC, BCL2 and PTEN. METTL3 depletion in AML cells reduces the translation of such transcripts, resulting in AKT activation, increased cell differentiation and apoptosis [43]. One recent study demonstrated a vital role of METTL3-regulated m^6^A modification in the maintenance and radioresistance of glioma stem-like cells [44]. METTL3 was upregulated in glioma stem-like cells over the matched differentiated glioma cells and its silencing suppressed tumor growth in vivo. SOX2, one of the glioma reprogramming factors [46], was methylated in its specific sites of mRNA 3′ UTR by METTL3. Together with further recruitment of HuR onto m^6^A-modified sites, SOX2 mRNA was stabilized. Consequently, SOX2 mediated the METTL3-dependent maintenance and radioresistance of glioma stem-like cells [44]. Another recent study reported that METTL3 was associated with chemo- and radioresistance in pancreatic cancer cells [45]. A sphere formation assay revealed that pancreatic cancer cells with METTL3 knockdown showed significantly lower self-renewal abilities than control cells. Moreover, METTL3 depletion enhanced chemo- and radiosensitivity of cancer cells, which suggests that METTL3 has an important role in the acquisition of resistance to anticancer drugs and irradiation [45]. 

Besides m^6^A methyltransferase activity, METTL3 has also been reported to interact with translation initiation machinery to promote translation of a subset of m^6^A containing mRNAs, independent of its catalytic activity or downstream m^6^A readers [47]. By recruiting eIF3 to the translation initiation complex in the cytoplasm, METTL3 enhanced translation of target mRNAs including two oncogenes, epidermal growth factor receptor (EGFR) and the Hippo pathway effector TAZ. Furthermore, METTL3 expression increased in lung adenocarcinoma, and it promoted growth, survival, and invasion of human lung cancer cells [47]. These findings shed light on a critical oncogenic role of METTL3 in carcinogenesis.

METTL14, another m^6^A writer protein, has also been linked to cancer development. In 2017, one study found that m^6^A modification decreased in hepatocellular carcinoma (HCC), especially in metastatic HCC, and METTL14 was responsible for the aberrant m^6^A modification in this kind of cancer [48]. It was also found that METTL14 knockdown raised the metastatic capacity of HCC, but METTL14 overexpression restrained invasiveness and metastasis in HCC. Mechanistically, METTL14-dependent m^6^A methylation promoted the recognition and binding of DGCR8 to pri-miR-126 and enhanced its processing to mature miR-126. Consequently, miR-126 mediated the suppressive effect of METTL14 in HCC metastasis [48]. However, another study showed that METTL3 was significantly upregulated in human HCC and high expression of METTL3 was associated with poor prognosis for HCC patients [49]. It was shown that depletion of METTL3 restrained HCC growth and metastasis both in vitro and in vivo, but overexpression of METTL3 enhanced HCC cell proliferation and migration, as well as tumor growth in vivo. SOCS2, a known tumor suppressor, was identified to be a downstream target of METTL3. METTL3 methylated SOCS2 mRNA and attenuated its stability by YTHDF2-dependent degradation pathway [49].

Recently, high expression of METTL14 was observed in acute myeloid leukemia (AML) cells with t(11q23), t(15;17), or t(8;21), as well as in hematopoietic stem/progenitor cells (HSPCs). However, expression of METTLl14 and m^6^A level decreased during myeloid differentiation of HSPCs and AML cells [50]. Moreover, METTL14 depletion inhibited AML cell proliferation or survival and promoted terminal myeloid differentiation of normal HSPCs and AML cells. Mechanistically, METTL14 plays a critical role in AML development and maintenance by directly methylating mRNA and regulating mRNA stability and translation. MYB and MYC, two known oncogenic transcription factors that contribute to AML development by inhibiting differentiation and promoting self-renewal of AML cells [51,52], are important targets of METTL14 in AML [50]. 

## 4. Function of m^6^A Erasers in Cancer Development

As the first identified RNA m^6^A eraser, FTO has been demonstrated to promote leukemic oncogene-mediated cell transformation and leukemogenesis [53]. FTO is activated by several leukemic oncoproteins, and therefore is highly expressed in several AML subtypes (e.g., t(11q23)/MLL-rearranged, t(15;17), FLT3-ITD, and/or NPM1-mutated AMLs). FTO not only promotes cell proliferation/transformation and inhibits apoptosis in vitro, but also significantly facilitates leukemogenesis in vivo. Furthermore, through negatively regulating a set of critical genes (e.g., ASB2 and RARA) in AML as RNA m^6^A demethylase, FTO plays an oncogenic role and inhibits all-trans-retinoic acid (ATRA)-mediated differentiation of leukemia cells [53]. The functional importance of FTO in tumor progression has also been revealed in lung squamous cell carcinoma (LUSC) [54]. MZF1-activated MYC expression has been reported to contribute to progression of lung adenocarcinoma [55]. FTO exerts its oncogenic function in LUSC by enhancing MZF1 expression, through reducing m^6^A levels and increasing the stability of MZF1 mRNA transcripts [54].

Recently, FTO was observed to be elevated in cervical squamous cell carcinoma (CSCC) tissue and promote the chemo-radiotherapy resistance of CSCC [56]. FTO positively regulates β-catenin expression by reducing m^6^A levels in its mRNA transcripts. Subsequently, excision repair cross-complementation group 1 (ERCC1), as a downstream effector of β-catenin, contributes to FTO/β-catenin-induced chemo-radiotherapy resistance in CSCC. Moreover, there is a positive correlation between FTO and β-catenin expression in human CSCC samples, and the combination of FTO and β-catenin confers better prognostic value for overall survival of CSCC than FTO alone [56]. Interestingly, one study links oncometabolite to m^6^A modification in cancer [57]. R-2-hydroxyglutarate (R-2HG), which is generated by mutant isocitrate dehydrogenase 1/2 (IDH1/2) and is regarded as an oncometabolite, also possesses anti-tumor effect in leukemia [57]. By directly binding and restraining the demethylase activity of FTO, R-2HG promotes overall m^6^A modification in R-2HG-sensitive leukemia cells. The stability of transcripts, such as MYC and CEBPA, is impaired by the R-2HG/FTO/m^6^A axis, likely by a YTHDF2-dependent degradation mechanism. Therefore, R-2HG displays anti-leukemia activity by suppressing FTO/m^6^A/MYC/CEBPA signaling, as well as relevant pathways [57]. 

ALKBH5-mediated demethylation of mRNA *N*^6^-methyladenosine has been linked to cancer stem cell phenotypes. The breast cancer stem cell (BCSC) phenotype is induced by hypoxia, which leads to a reduction in total RNA m^6^A by activating the expression of ALKBH5 in an HIF-dependent manner [58]. NANOG, a pluripotency factor functioning in the maintenance and specification of cancer stem cells, is demethylated by ALKBH5 at an m^6^A residue in the 3′ UTR. As a result, NANOG mRNA is stabilized, and upregulated NANOG contributes to the enhanced percentage of BCSCs under hypoxic conditions [58]. 

Additionally, ALKBH5 maintains tumorigenicity of glioblastoma stem-like cells (GSCs) [59]. ALKBH5 is required for GSC self-renewal and predicts poor survival of glioblastoma patients. Furthermore, ALKBH5 demethylates FOXM1 pre-mRNA and increases its stability by interaction with HuR [60]. Subsequently, upregulated FOXM1 mediates ALKBH5-dependent GSC proliferation, self-renewal, and tumorigenicity [59]. Recently, another study also reported that RNA m^6^A modification regulated the self-renewal and tumorigenesis of glioblastoma stem cells [61]. It was found that knockdown of METTL3 or METTL14 expression enhanced, but overexpression of METTL3 inhibited, GSC growth and self-renewal. Likewise, inhibition of the RNA demethylase FTO with its inhibitor MA2 reduced GSC-initiated tumor growth and prolonged the lifespan of GSC-grafted mice substantially [61]. 

## 5. Function of m^6^A Readers in Cancer Development

As an m^6^A reader, YTHDF2 can selectively recognize and bind to m^6^A sites to mediate mRNA degradation [17]. One study demonstrated that miR-145 could target the 3′ UTR of YTHDF2 mRNA and suppress the expression of YTHDF2 at the levels of mRNA and protein in hepatocellular cells, leading to reduced mRNA degradation and increased mRNA m^6^A levels [62]. Moreover, YTHDF2 expression detected by immunohistochemical staining showed that it was closely related to malignancy of HCC, which indicates that it may play an important oncogenic role in liver cancer progression [62]. As another cytoplasmic m^6^A reader, YTHDF1 has been reported to be associated with poor prognosis in patients with hepatocellular carcinoma [63]. Based on The Cancer Genome Atlas (TCGA) data, researchers found that YTHDF1 was significantly enhanced in HCC and was positively correlated with pathology stage. In addition, Kaplan-Meier analysis showed that higher YTHDF1 expression was associated with worse survival of HCC patients [63]. 

IGF2BPs (IGF2BP1/2/3) are a new family of m^6^A reader proteins that directly bind mRNA transcripts via the consensus GG(m^6^A)C sequence [42] (Figure 1). On the one hand, IGF2BPs can increase the stability of m^6^A-modified mRNAs in the nucleus by recruiting HuR and MATR3, two known mRNA stabilizers. On the other hand, IGF2BPs enhance the translation of m^6^A-containing mRNAs in the cytoplasm. Moreover, IGF2BPs play an oncogenic role in human cancer cells by their oncogenic transcript targets, such as *MYC* [42]. However, YTHDC2 and hnRNPA2B1, two other m^6^A reader proteins, have not been documented for their involvement in m^6^A dysfunction in cancer. In view of their confirmed oncogenic role in tumor [64,65,66], their regulatory function in m^6^A-related cancer progression is deducible, but further experimental verification is still required. 

## 6. Other Protein Factors Involved in Regulation of m^6^A Modification in Cancer Development

In addition to m^6^A writers, erasers, and readers, several protein factors have also been identified to modulate m^6^A modification and correlate with cancer development. Zinc finger protein 217 (ZFP217) is a transcription factor that directly activates the transcription of core stem cell genes and regulates the pluripotency of embryonic stem cells and somatic cell reprogramming [67]. In addition, by interacting with METTL3 and eliminating its RNA methyltransferases activity, ZFP217 prevents the deposition of m^6^A at transcripts and enhances their stabilization, including mRNAs of core stem-cell genes *Nanog*, *Sox2*, *Klf4*, and *c-Myc* [67]. Considering the accumulating evidence that ZNF217 plays an essential role in tumor progression, metastasis, and chemoresistance [68,69], it is predictable that ZNF217-regulated m^6^A modification, possibly by interaction with METTL3, is relevant in human cancers. Recently, it was discovered that the oncoprotein hepatitis B X-interacting protein (HBXIP), whose aberrant expression drives the aggressiveness of breast cancer, releases the expression of METTL3 by repressing its inhibitor let-7 g [70]. Interestingly, METTL3 in return activates the expression of HBXIP through m^6^A modification. Therefore, mutual regulation between HBXIP and METTL3 promotes progression of breast cancer [70]. Another recent study revealed that SMAD2/3, members of the TGFβ signaling pathway, which is associated with tumor progression [71], promoted binding of the m^6^A methyltransferase complex to a subset of mRNA transcripts and caused their destabilization and rapid degradation [72]. Thus, the TGFβ pathway may be involved in cancer development by affecting m^6^A epigenetic modification. In sum, these protein factors may participate in cancer progression by regulating expression or function of m^6^A methyltransferases.

## 7. Conclusions 

Multiple lines of evidence show that m^6^A modification of mRNA is deregulated in numerous cancers, and its role in cancers has been verified by both in vitro and in vivo studies. Clarifying the molecular mechanisms that mediate these m^6^A modification changes in RNA and identifying the aberrant expression of m^6^A regulatory factors in clinical biopsy specimens could contribute largely to early diagnosis of cancer, prediction of cancer prognosis, and provision of novel therapeutic approaches for cancer. We summarize here that not only m^6^A writers [43,44,48,49,50], erasers [53,54,56,57,58,59], and readers [42,62,63], but also other protein factors, including oncoprotein [70], transcription factor [67], and signal transduction factor [72], affect m^6^A abundance and function in various cancers (Table 1). Therefore, overexpression or depletion of these m^6^A-related factors may alter m^6^A modification in tumors and interfere with cancer progression. 

Notably, as shown in Table 1, the majority of m^6^A regulatory factors play an oncogenic role in cancers, despite m^6^A writers and erasers performing opposite functions in m^6^A modification. As one striking example, both m^6^A writer METTL14 [50] and m^6^A eraser FTO [53] are found to be highly expressed in AMLs, even in the same subtype—as in t(11q23) and t(15;17)—and play critical roles in AML development. One reasonable interpretation of these paradoxical phenomena may be that the difference in their protein structures determines their substrate specificity [73,74]. By recognizing and targeting different mRNA targets, including oncogenes and tumor suppressors, m^6^A writers and erasers can exert an analogous function in the development of the same cancer type. Additionally, it cannot be overlooked that different reader proteins bind the m^6^A of mRNA targets and confer divergent or even opposite final status. Therefore, developing an effective therapeutic strategy for one cancer type should be based on an overall consideration of the m^6^A writers’ and erasers’ functions and downstream targets, as well as m^6^A readers’ binding to the targets. 

Recently, inhibitors of m^6^A-modifying enzymes have also been explored and identified as changing cellular m^6^A abundance. For example, meclofenamic acid was found to be a highly selective inhibitor of FTO in vivo for competing on FTO binding to m^6^A-containing nucleic acid [75]. However, some drugs are suitable for only one specific type of cancer, but not for multiple cancers, because of the heterogeneity of cancer [76]. Consequently, more clinically applicable selective and powerful drugs targeting regulatory proteins of m^6^A are expected to be developed. Elucidating the regulatory mechanism of mRNA m^6^A modification in carcinogenesis and cancer progression would serve to develop m^6^A-related factors as valuable targets for the treatment of human cancers.

## Figures and Tables

**Table 1:** Function of m^6^A regulatory factors in various cancers.

Type	Molecule	Cancer	Role	Function	Reference
m^6^A writer	METTL3	hepatocellular carcinoma	oncogenic	Attenuate SOCS2 mRNA stability	[49]
	METTL3	glioma	oncogenic	Methylate and stabilize SOX2 mRNA	[44]
	METTL3	acute myeloid leukemia	oncogenic	Control expression of c-Myc, Bcl-2 and PTEN	[43]
	METTL14	acute myeloid leukemia	oncogenic	Regulate mRNA stability and translation of MYB and MYC	[50]
	METTL14	hepatocellular carcinoma	tumor suppressive	Regulate processing of miR-126 by DGCR8	[48]
m^6^A eraser	FTO	acute myeloid leukemia	oncogenic	Target a set of critical genes including ASB2 and RARA	[53]
	FTO	leukemia	oncogenic	Increase stability of Myc/CEBPA transcripts	[57]
	FTO	cervical squamous cell carcinoma	oncogenic	Positively regulate β-catenin/ERCC1 axis	[56]
	FTO	lung cancer	oncogenic	Demethylate MZF1 mRNA and increase its stability	[54]
	ALKBH5	breast cancer	oncogenic	Demethylate NANOG and increase its mRNA level	[58]
	ALKBH5	glioblastoma	oncogenic	Demethylate FOXM1 that promotes GSC tumorigenicity	[59]
m^6^A reader	YTHDF1	hepatocellular carcinoma	oncogenic	Associated with poor prognosis of HCC patients	[63]
	YTHDF2	hepatocellular carcinoma	oncogenic	Associated with malignancy of cancer	[62]
	IGF2BP1/2/3	cervical cancer, liver cancer	oncogenic	Stabilize methylated mRNAs of oncogenic targets such as MYC	[42]
Protein factor	ZFP217	N/A	oncogenic	Interact with METTL3 and sequester it into an inactive complex	[67]
	SMAD2/3	N/A	N/A	Promote binding of m^6^A methyltransferase complex to mRNA transcripts	[72]
	HBXIP	breast cancer	oncogenic	Upregulate METTL3 by inhibiting let-7 g	[70]

## References

[1] He C. (2010). Grand challenge commentary: RNA epigenetics?. Nat. Chem. Biol..

[2] Niu Y., Zhao X., Wu Y.S., Li M.M., Wang X.J., Yang Y.G. (2013). *N*^6^-methyl-adenosine (m^6^A) in RNA: An old modification with a novel epigenetic function. Genom. Proteom. Bioinform..

[3] Roundtree I.A., Evans M.E., Pan T., He C. (2017). Dynamic RNA Modifications in Gene Expression Regulation. Cell.

[4] Desrosiers R., Friderici K., Rottman F. (1974). Identification of methylated nucleosides in messenger RNA from Novikoff hepatoma cells. Proc. Natl. Acad. Sci. USA.

[5] Dominissini D., Moshitch-Moshkovitz S., Schwartz S., Salmon-Divon M., Ungar L., Osenberg S., Cesarkas K., Jacob-Hirsch J., Amariglio N., Kupiec M. (2012). Topology of the human and mouse m^6^A RNA methylomes revealed by m^6^A-seq. Nature.

[6] Meyer K.D., Saletore Y., Zumbo P., Elemento O., Mason C.E., Jaffrey S.R. (2012). Comprehensive analysis of mRNA methylation reveals enrichment in 3’ UTRs and near stop codons. Cell.

[7] Dominissini D., Moshitch-Moshkovitz S., Salmon-Divon M., Amariglio N., Rechavi G. (2013). Transcriptome-wide mapping of *N*(6)-methyladenosine by m(6)A-seq based on immunocapturing and massively parallel sequencing. Nat. Protoc..

[8] Liu N., Dai Q., Zheng G., He C., Parisien M., Pan T. (2015). *N*(6)-methyladenosine-dependent RNA structural switches regulate RNA-protein interactions. Nature.

[9] Liu N., Zhou K.I., Parisien M., Dai Q., Diatchenko L., Pan T. (2017). *N*^6^-methyladenosine alters RNA structure to regulate binding of a low-complexity protein. Nucleic Acids Res..

[10] Xiao W., Adhikari S., Dahal U., Chen Y.S., Hao Y.J., Sun B.F., Sun H.Y., Li A., Ping X.L., Lai W.Y. (2016). Nuclear m(6)A Reader YTHDC1 Regulates mRNA Splicing. Mol. Cell.

[11] Zhao X., Yang Y., Sun B.F., Shi Y., Yang X., Xiao W., Hao Y.J., Ping X.L., Chen Y.S., Wang W.J. (2014). FTO-dependent demethylation of *N*^6^-methyladenosine regulates mRNA splicing and is required for adipogenesis. Cell Res..

[12] Zheng G., Dahl J.A., Niu Y., Fedorcsak P., Huang C.M., Li C.J., Vagbo C.B., Shi Y., Wang W.L., Song S.H. (2013). ALKBH5 is a mammalian RNA demethylase that impacts RNA metabolism and mouse fertility. Mol. Cell.

[13] Fustin J.M., Doi M., Yamaguchi Y., Hida H., Nishimura S., Yoshida M., Isagawa T., Morioka M.S., Kakeya H., Manabe I. (2013). RNA-methylation-dependent RNA processing controls the speed of the circadian clock. Cell.

[14] Roundtree I.A., Luo G.Z., Zhang Z., Wang X., Zhou T., Cui Y., Sha J., Huang X., Guerrero L., Xie P. (2017). YTHDC1 mediates nuclear export of *N*(6)-methyladenosine methylated mRNAs. eLife.

[15] Wang X., Zhao B.S., Roundtree I.A., Lu Z., Han D., Ma H., Weng X., Chen K., Shi H., He C. (2015). *N*(6)-methyladenosine Modulates Messenger RNA Translation Efficiency. Cell.

[16] Li A., Chen Y.S., Ping X.L., Yang X., Xiao W., Yang Y., Sun H.Y., Zhu Q., Baidya P., Wang X. (2017). Cytoplasmic m(6)A reader YTHDF3 promotes mRNA translation. Cell Res..

[17] Wang X., Lu Z., Gomez A., Hon G.C., Yue Y., Han D., Fu Y., Parisien M., Dai Q., Jia G. (2014). *N*6-methyladenosine-dependent regulation of messenger RNA stability. Nature.

[18] Du H., Zhao Y., He J., Zhang Y., Xi H., Liu M., Ma J., Wu L. (2016). YTHDF2 destabilizes m(6)A-containing RNA through direct recruitment of the CCR4-NOT deadenylase complex. Nat. Commun..

[19] Zhao B.S., Roundtree I.A., He C. (2017). Post-transcriptional gene regulation by mRNA modifications. Nat. Rev. Mol. Cell. Biol..

[20] Xiang J.F., Yang Q., Liu C.X., Wu M., Chen L.L., Yang L. (2018). *N*(6)-Methyladenosines Modulate A-to-I RNA Editing. Mol. Cell.

[21] Hsu P.J., Zhu Y., Ma H., Guo Y., Shi X., Liu Y., Qi M., Lu Z., Shi H., Wang J. (2017). Ythdc2 is an *N*(6)-methyladenosine binding protein that regulates mammalian spermatogenesis. Cell Res..

[22] Wang Y., Li Y., Toth J.I., Petroski M.D., Zhang Z., Zhao J.C. (2014). *N*^6^-methyladenosine modification destabilizes developmental regulators in embryonic stem cells. Nat. Cell Biol..

[23] Batista P.J., Molinie B., Wang J., Qu K., Zhang J., Li L., Bouley D.M., Lujan E., Haddad B., Daneshvar K. (2014). m(6)A RNA modification controls cell fate transition in mammalian embryonic stem cells. Cell Stem Cell.

[24] Chen T., Hao Y.J., Zhang Y., Li M.M., Wang M., Han W., Wu Y., Lv Y., Hao J., Wang L. (2015). m(6)A RNA methylation is regulated by microRNAs and promotes reprogramming to pluripotency. Cell Stem Cell.

[25] Geula S., Moshitch-Moshkovitz S., Dominissini D., Mansour A.A., Kol N., Salmon-Divon M., Hershkovitz V., Peer E., Mor N., Manor Y.S. (2015). Stem cells. m^6^A mRNA methylation facilitates resolution of naive pluripotency toward differentiation. Science.

[26] Yoon K.J., Ringeling F.R., Vissers C., Jacob F., Pokrass M., Jimenez-Cyrus D., Su Y., Kim N.S., Zhu Y., Zheng L. (2017). Temporal Control of Mammalian Cortical Neurogenesis by m(6)A Methylation. Cell.

[27] Tuck M.T., James C.B., Kelder B., Kopchick J.J. (1996). Elevation of internal 6-methyladenine mRNA methyltransferase activity after cellular transformation. Cancer Lett..

[28] Huang W., Qi C.B., Lv S.W., Xie M., Feng Y.Q., Huang W.H., Yuan B.F. (2016). Determination of DNA and RNA Methylation in Circulating Tumor Cells by Mass Spectrometry. Anal. Chem..

[29] Ni T.K., Elman J.S., Jin D.X., Gupta P.B., Kuperwasser C. (2018). Premature polyadenylation of MAGI3 is associated with diminished *N*(6)-methyladenosine in its large internal exon. Sci. Rep..

[30] Dai D., Wang H., Zhu L., Jin H., Wang X. (2018). *N*^6^-methyladenosine links RNA metabolism to cancer progression. Cell Death Dis..

[31] Pan Y., Ma P., Liu Y., Li W., Shu Y. (2018). Multiple functions of m(6)A RNA methylation in cancer. J. Hematol. Oncol..

[32] Chi H.C., Tsai C.Y., Tsai M.M., Lin K.H. (2018). Impact of DNA and RNA Methylation on Radiobiology and Cancer Progression. Int. J. Mol. Sci..

[33] Bokar J.A., Shambaugh M.E., Polayes D., Matera A.G., Rottman F.M. (1997). Purification and cDNA cloning of the AdoMet-binding subunit of the human mRNA (*N*^6^-adenosine)-methyltransferase. RNA.

[34] Liu J., Yue Y., Han D., Wang X., Fu Y., Zhang L., Jia G., Yu M., Lu Z., Deng X. (2014). A METTL3-METTL14 complex mediates mammalian nuclear RNA *N*^6^-adenosine methylation. Nat. Chem. Biol..

[35] Ping X.L., Sun B.F., Wang L., Xiao W., Yang X., Wang W.J., Adhikari S., Shi Y., Lv Y., Chen Y.S. (2014). Mammalian WTAP is a regulatory subunit of the RNA *N*^6^-methyladenosine methyltransferase. Cell Res..

[36] Warda A.S., Kretschmer J., Hackert P., Lenz C., Urlaub H., Hobartner C., Sloan K.E., Bohnsack M.T. (2017). Human METTL16 is a *N*(6)-methyladenosine (m(6)A) methyltransferase that targets pre-mRNAs and various non-coding RNAs. EMBO Rep..

[37] Frayling T.M., Timpson N.J., Weedon M.N., Zeggini E., Freathy R.M., Lindgren C.M., Perry J.R., Elliott K.S., Lango H., Rayner N.W. (2007). A common variant in the FTO gene is associated with body mass index and predisposes to childhood and adult obesity. Science.

[38] Jia G., Fu Y., Zhao X., Dai Q., Zheng G., Yang Y., Yi C., Lindahl T., Pan T., Yang Y.G. (2011). *N*^6^-methyladenosine in nuclear RNA is a major substrate of the obesity-associated FTO. Nat. Chem. Biol..

[39] Shi H., Wang X., Lu Z., Zhao B.S., Ma H., Hsu P.J., Liu C., He C. (2017). YTHDF3 facilitates translation and decay of *N*(6)-methyladenosine-modified RNA. Cell Res..

[40] Alarcon C.R., Goodarzi H., Lee H., Liu X., Tavazoie S., Tavazoie S.F. (2015). HNRNPA2B1 Is a Mediator of m(6)A-Dependent Nuclear RNA Processing Events. Cell.

[41] Meyer K.D., Patil D.P., Zhou J., Zinoviev A., Skabkin M.A., Elemento O., Pestova T.V., Qian S.B., Jaffrey S.R. (2015). 5’ UTR m(6)A Promotes Cap-Independent Translation. Cell.

[42] Huang H., Weng H., Sun W., Qin X., Shi H., Wu H., Zhao B.S., Mesquita A., Liu C., Yuan C.L. (2018). Recognition of RNA *N*(6)-methyladenosine by IGF2BP proteins enhances mRNA stability and translation. Nat. Cell Biol..

[43] Huang H., Weng H., Sun W., Qin X., Vu L.P., Pickering B.F., Cheng Y., Zaccara S., Nguyen D., Minuesa G. (2017). The *N*(6)-methyladenosine (m(6)A)-forming enzyme METTL3 controls myeloid differentiation of normal hematopoietic and leukemia cells. Nat. Med..

[44] Visvanathan A., Patil V., Arora A., Hegde A.S., Arivazhagan A., Santosh V., Somasundaram K. (2018). Essential role of METTL3-mediated m(6)A modification in glioma stem-like cells maintenance and radioresistance. Oncogene.

[45] Taketo K., Konno M., Asai A., Koseki J., Toratani M., Satoh T., Doki Y., Mori M., Ishii H., Ogawa K. (2018). The epitranscriptome m^6^A writer METTL3 promotes chemo- and radioresistance in pancreatic cancer cells. Int. J. Oncol..

[46] Suva M.L., Rheinbay E., Gillespie S.M., Patel A.P., Wakimoto H., Rabkin S.D., Riggi N., Chi A.S., Cahill D.P., Nahed B.V. (2014). Reconstructing and reprogramming the tumor-propagating potential of glioblastoma stem-like cells. Cell.

[47] Lin S., Choe J., Du P., Triboulet R., Gregory R.I. (2016). The m(6)A Methyltransferase METTL3 Promotes Translation in Human Cancer Cells. Mol. Cell.

[48] Ma J.Z., Yang F., Zhou C.C., Liu F., Yuan J.H., Wang F., Wang T.T., Xu Q.G., Zhou W.P., Sun S.H. (2017). METTL14 suppresses the metastatic potential of hepatocellular carcinoma by modulating *N*(6)-methyladenosine-dependent primary MicroRNA processing. Hepatology.

[49] Chen M., Wei L., Law C.T., Tsang F.H., Shen J., Cheng C.L., Tsang L.H., Ho D.W., Chiu D.K., Lee J.M. (2018). RNA *N*^6^-methyladenosine methyltransferase-like 3 promotes liver cancer progression through YTHDF2-dependent posttranscriptional silencing of SOCS2. Hepatology.

[50] Weng H., Huang H., Wu H., Qin X., Zhao B.S., Dong L., Shi H., Skibbe J., Shen C., Hu C. (2018). METTL14 Inhibits Hematopoietic Stem/Progenitor Differentiation and Promotes Leukemogenesis via mRNA m(6)A Modification. Cell Stem Cell.

[51] Wall M., Poortinga G., Hannan K.M., Pearson R.B., Hannan R.D., McArthur G.A. (2008). Translational control of c-MYC by rapamycin promotes terminal myeloid differentiation. Blood.

[52] Zhao L., Ye P., Gonda T.J. (2014). The MYB proto-oncogene suppresses monocytic differentiation of acute myeloid leukemia cells via transcriptional activation of its target gene GFI1. Oncogene.

[53] Li Z., Weng H., Su R., Weng X., Zuo Z., Li C., Huang H., Nachtergaele S., Dong L., Hu C. (2017). FTO Plays an Oncogenic Role in Acute Myeloid Leukemia as a *N*(6)-Methyladenosine RNA Demethylase. Cancer Cell.

[54] Liu J., Ren D., Du Z., Wang H., Zhang H., Jin Y. (2018). m(6)A demethylase FTO facilitates tumor progression in lung squamous cell carcinoma by regulating MZF1 expression. Biochem. Biophys. Res. Commun..

[55] Tsai L.H., Wu J.Y., Cheng Y.W., Chen C.Y., Sheu G.T., Wu T.C., Lee H. (2015). The MZF1/c-MYC axis mediates lung adenocarcinoma progression caused by wild-type lkb1 loss. Oncogene.

[56] Zhou S., Bai Z.L., Xia D., Zhao Z.J., Zhao R., Wang Y.Y., Zhe H. (2018). FTO regulates the chemo-radiotherapy resistance of cervical squamous cell carcinoma (CSCC) by targeting beta-catenin through mRNA demethylation. Mol. Carcinog..

[57] Su R., Dong L., Li C., Nachtergaele S., Wunderlich M., Qing Y., Deng X., Wang Y., Weng X., Hu C. (2018). R-2HG Exhibits Anti-tumor Activity by Targeting FTO/m(6)A/MYC/CEBPA Signaling. Cell.

[58] Zhang C., Samanta D., Lu H., Bullen J.W., Zhang H., Chen I., He X., Semenza G.L. (2016). Hypoxia induces the breast cancer stem cell phenotype by HIF-dependent and ALKBH5-mediated m(6)A-demethylation of NANOG mRNA. Proc. Natl. Acad. Sci. USA.

[59] Zhang S., Zhao B.S., Zhou A., Lin K., Zheng S., Lu Z., Chen Y., Sulman E.P., Xie K., Bogler O. (2017). m(6)A Demethylase ALKBH5 Maintains Tumorigenicity of Glioblastoma Stem-like Cells by Sustaining FOXM1 Expression and Cell Proliferation Program. Cancer Cell.

[60] Dixit D., Xie Q., Rich J.N., Zhao J.C. (2017). Messenger RNA Methylation Regulates Glioblastoma Tumorigenesis. Cancer Cell.

[61] Cui Q., Shi H., Ye P., Li L., Qu Q., Sun G., Sun G., Lu Z., Huang Y., Yang C.G. (2017). m(6)A RNA Methylation Regulates the Self-Renewal and Tumorigenesis of Glioblastoma Stem Cells. Cell Rep..

[62] Yang Z., Li J., Feng G., Gao S., Wang Y., Zhang S., Liu Y., Ye L., Li Y., Zhang X. (2017). MicroRNA-145 Modulates *N*(6)-Methyladenosine Levels by Targeting the 3’-Untranslated mRNA Region of the *N*(6)-Methyladenosine Binding YTH Domain Family 2 Protein. J. Biol. Chem..

[63] Zhao X., Chen Y., Mao Q., Jiang X., Jiang W., Chen J., Xu W., Zhong L., Sun X. (2018). Overexpression of YTHDF1 is associated with poor prognosis in patients with hepatocellular carcinoma. Cancer Biomark..

[64] Tanabe A., Tanikawa K., Tsunetomi M., Takai K., Ikeda H., Konno J., Torigoe T., Maeda H., Kutomi G., Okita K. (2016). RNA helicase YTHDC2 promotes cancer metastasis via the enhancement of the efficiency by which HIF-1alpha mRNA is translated. Cancer Lett..

[65] Golan-Gerstl R., Cohen M., Shilo A., Suh S.S., Bakacs A., Coppola L., Karni R. (2011). Splicing factor hnRNP A2/B1 regulates tumor suppressor gene splicing and is an oncogenic driver in glioblastoma. Cancer Res..

[66] Barcelo C., Etchin J., Mansour M.R., Sanda T., Ginesta M.M., Sanchez-Arevalo Lobo V.J., Real F.X., Capella G., Estanyol J.M., Jaumot M. (2014). Ribonucleoprotein HNRNPA2B1 interacts with and regulates oncogenic KRAS in pancreatic ductal adenocarcinoma cells. Gastroenterology.

[67] Aguilo F., Zhang F., Sancho A., Fidalgo M., Di Cecilia S., Vashisht A., Lee D.F., Chen C.H., Rengasamy M., Andino B. (2015). Coordination of m(6)A mRNA Methylation and Gene Transcription by ZFP217 Regulates Pluripotency and Reprogramming. Cell Stem Cell.

[68] Thollet A., Vendrell J.A., Payen L., Ghayad S.E., Ben Larbi S., Grisard E., Collins C., Villedieu M., Cohen P.A. (2010). ZNF217 confers resistance to the pro-apoptotic signals of paclitaxel and aberrant expression of Aurora-A in breast cancer cells. Mol. Cancer.

[69] Littlepage L.E., Adler A.S., Kouros-Mehr H., Huang G., Chou J., Krig S.R., Griffith O.L., Korkola J.E., Qu K., Lawson D.A. (2012). The transcription factor ZNF217 is a prognostic biomarker and therapeutic target during breast cancer progression. Cancer Discov..

[70] Cai X., Wang X., Cao C., Gao Y., Zhang S., Yang Z., Liu Y., Zhang X., Zhang W., Ye L. (2018). HBXIP-elevated methyltransferase METTL3 promotes the progression of breast cancer via inhibiting tumor suppressor let-7 g. Cancer Lett..

[71] David C.J., Massague J. (2018). Contextual determinants of TGFβ action in development, immunity and cancer. Nat. Rev. Mol. Cell Biol..

[72] Bertero A., Brown S., Madrigal P., Osnato A., Ortmann D., Yiangou L., Kadiwala J., Hubner N.C., de Los Mozos I.R., Sadee C. (2018). The SMAD2/3 interactome reveals that TGFβ controls m(6)A mRNA methylation in pluripotency. Nature.

[73] Han Z., Niu T., Chang J., Lei X., Zhao M., Wang Q., Cheng W., Wang J., Feng Y., Chai J. (2010). Crystal structure of the FTO protein reveals basis for its substrate specificity. Nature.

[74] Wang X., Feng J., Xue Y., Guan Z., Zhang D., Liu Z., Gong Z., Wang Q., Huang J., Tang C. (2016). Structural basis of *N*(6)-adenosine methylation by the METTL3-METTL14 complex. Nature.

[75] Huang Y., Yan J., Li Q., Li J., Gong S., Zhou H., Gan J., Jiang H., Jia G.F., Luo C. (2015). Meclofenamic acid selectively inhibits FTO demethylation of m^6^A over ALKBH5. Nucleic Acids Res..

[76] Dagogo-Jack I., Shaw A.T. (2018). Tumour heterogeneity and resistance to cancer therapies. Nat. Rev. Clin. Oncol..

